# Endocannabinoid System and Migraine Pain: An Update

**DOI:** 10.3389/fnins.2018.00172

**Published:** 2018-03-19

**Authors:** Rosaria Greco, Chiara Demartini, Anna M. Zanaboni, Daniele Piomelli, Cristina Tassorelli

**Affiliations:** ^1^Laboratory of Neurophysiology of Integrative Autonomic Systems, Headache Science Centre, IRCCS Mondino Foundation, Pavia, Italy; ^2^Department of Brain and Behavioral Sciences, University of Pavia, Pavia, Italy; ^3^Department of Anatomy and Neurobiology, University of California, Irvine, Irvine, CA, United States

**Keywords:** migraine, inflammation, trigeminal hyperalgesia, endocannabinoid system, FAAH inhibitors

## Abstract

The trigeminovascular system (TS) activation and the vasoactive release from trigeminal endings, in proximity of the meningeal vessels, are considered two of the main effector mechanisms of migraine attacks. Several other structures and mediators are involved, however, both upstream and alongside the TS. Among these, the endocannabinoid system (ES) has recently attracted considerable attention. Experimental and clinical data suggest indeed a link between dysregulation of this signaling complex and migraine headache. Clinical observations, in particular, show that the levels of anandamide (AEA)—one of the two primary endocannabinoid lipids—are reduced in cerebrospinal fluid and plasma of patients with chronic migraine (CM), and that this reduction is associated with pain facilitation in the spinal cord. AEA is produced on demand during inflammatory conditions and exerts most of its effects by acting on cannabinoid (CB) receptors. AEA is rapidly degraded by fatty acid amide hydrolase (FAAH) enzyme and its levels can be modulated in the peripheral and central nervous system (CNS) by FAAH inhibitors. Inhibition of AEA degradation via FAAH is a promising therapeutic target for migraine pain, since it is presumably associated to an increased availability of the endocannabinoid, specifically at the site where its formation is stimulated (e.g., trigeminal ganglion and/or meninges), thus prolonging its action.

## Introduction

Migraine is one of the most disabling painful conditions and a very common disorder (Global Burden of Disease, 2015). Although the pathophysiology of migraine is still largely elusive, the trigeminovascular system (TS) activation and the neurogenic inflammation of the dura mater are widely recognized as two key mechanisms underlying migraine attacks (Moskowitz, 1993). TS activation causes neuropeptide release from trigeminal endings in proximity of the meningeal vessels. Meningeal release of mediators produces peripheral sensitization, which is aggravated by central sensitization when the attacks recur more frequently. Calcitonin gene-related peptide (CGRP) and other inflammatory mediators, released from sensory nerve terminals (Moskowitz, 1993), irritate and further dilate blood vessels, thus resulting in the release of additional neuropeptides from the sensory neurons and an increase of pain impulses that are transmitted to the nucleus trigeminalis caudalis (NTC). The activated NTC relays in turn pain signals to higher brain centers, including thalamus and cortex. In this circuitry, another interesting player is nitric oxide (NO), which contributes to the perivascular sensory afferent nerve fibers activation in the meninges and to neuropeptides release or NO formation by neuronal NO synthase (nNOS) (Messlinger et al., 2000; Alaşehirli et al., 2013; Ramachandran et al., 2014). Evidence suggests that the origin of migraine attacks is the interaction of internal or external triggers with dysfunctional central structures (brainstem, thalamus) involved in the transmission and regulation of pain sensation (Goadsby, 2002; Knight et al., 2005; Coppola et al., 2013).

Current standards of care for migraine have a moderate effectiveness at best and, in some cases, limited tolerability. Specifically, prophylactic treatments (beta blockers, antiepileptic drugs) may induce weight gain, depression, behavioral or cognitive disturbances. Triptans, 5-HT1-Receptor agonists, used for acute treatment, may cause a serious long-term side effects such us chest pain, neck and limbs with paresthesias and hot or cold sensations. Analgesics combinations and non-steroidal anti-inflammatory drugs, for acute migraine can lead to gastrointestinal and cardio-renal side effects (Antonaci et al., 2016). The endocannabinoid system (ES) has recently received attention in regard to pain control, after the availability of probes capable of modulating its activity via the interaction with endocannabinoid catabolic enzymes (Chiou et al., 2013). In this review, we summarize results collected in studies aimed at evaluating the role of the endocannabinoids (ECs) in migraine, with a specific focus on fatty acid amide hydrolase (FAAH) inhibitors.

## Endocannabinoid signaling

EC signaling in the nervous system is different from those of the classic neurotransmission systems, where the depolarization of the presynaptic neuron causes neurotransmitters release which in turn activates their receptors on the postsynaptic neuron. The ECs act as retrograde or local neurotransmitters, and are produced and released from neurons upon demand. They bind to CB1-type cannabinoid (CB_1_) receptors, which are localized on presynaptic terminals of excitatory and inhibitory neurons throughout the brain and spinal cord (Alger and Kim, 2011). CB_1_ receptors are seven trans-membrane domain proteins that belong to the rhodopsin family of G protein-coupled receptors, specifically those of the Gi/o family (GPCRs). Recent crystallographic studies reported that extracellular surface of CB1 receptor is different from other lipid-activated GPCRs with a critical part of the ligand binding pocket (Hua et al., 2016; Shao et al., 2016). CB_1_ receptors are found in neuroanatomical regions involved in pain processing, and inhibit the release of neurotransmitters such as γ-aminobutyric acid, glutamate, dopamine, noradrenaline and acetylcholine (Katona and Freund, 2008). Though abundant in neurons of the central nervous system (CNS), CB_1_ receptors are also expressed in peripheral neurons and many non-neural cells (Kendall and Yudowski, 2017). A second cannabinoid receptor subtype, CB_2_, is found primarily in immune cells (Gerdeman et al., 2002). Furthermore AEA and 2-arachidonoylglycerol (2-AG), the best characterized ECs, are produced in structures involved in nociception, such as the skin, dorsal root ganglia, spinal cord, periaqueductal gray matter (PAG), and rostral ventromedial medulla (RVM) (Katona and Freund, 2008). Through activation of CB_1_ receptors, AEA and 2-AG can influence a variety of physiological processes, including energy balance, cognition and pain (Bellocchio et al., 2008; Kano et al., 2009).

In neurons, as in other cells, the ECs are not stored in vesicles but are enzymatically produced upon demand from membrane glycerophospholipid precursors. Enzymes involved in AEA and 2-AG formation are N-acylphosphatidylethanolamine-phospholipase D (NAPE-PLD) and diacylglycerol lipase (DGL), respectively (Bisogno et al., 2003; Okamoto et al., 2007). However, other pathways through which AEA can be produced have been described (Liu et al., 2006; Jin et al., 2007). Moreover, several enzymes involved in ECs biosynthesis, such as NAPE-PLD, give rise not only to AEA but also to structurally similar lipid messengers that do not bind and activate CB_1_, i.e., oleoylethanolamide (OEA) and palmitoylethanolamide (PEA) (Gaetani et al., 2010). AEA acts primarily on CB_1_ receptors, though pharmacological actions on other receptors, such as transient receptor potential (TRP) V1, have been described (Puente et al., 2011), TRPV2, TRPA1, and TRPM8 (Di Marzo and De Petrocellis, 2010).

2-AG production also occurs via multiple biosynthetic pathways, in which diacylglycerol (DAG), produced by the action of either phospholipase C (PLC) or phosphatidic acid phosphohydrolase, acts as a common precursor. DAG is transformed into 2-AG by DGL; alternatively, phospholipase A_1_ may convert phosphatidyl-inositol into lyso-phosphatidyl-inositol, which may be transformed to 2-AG by PLC.

The ECs are quickly deactivated by uptake into cells followed by intracellular hydrolysis (Urquhart et al., 2015). Transporter proteins remove AEA from the extracellular space; successively AEA is mostly degraded by FAAH, releasing arachidonic acid (AA) and ethanolamine. 2-AG is hydrolyzed mainly by the serine hydrolase, monoacylglycerol lipase (MGL), which produces AA and glycerol. However, it may be also degraded by α,β-hydrolase-6 or converted to bioactive oxygenated products by COX2. Thus, the enzymes responsible for the biosynthetic, as well as degradative pathways are essential in the regulation and modulation of EC levels in the CNS. Moreover, differential cellular distribution of the synthesizing and degrading enzymes may control of EC activity. Thus, selective pharmacological or genetic manipulations of FAAH and MGL activities can be used to evaluate the functions of each EC in animal model.

## Relationship between ES dysregulation and migraine: human and experimental studies

The ES may modulate the cerebrovascular tone, through interaction with serotonergic system, NO synthesis, and neuropeptides release (Pertwee, 2001), neurotransmitters that play a crucial role in migraine pathogenesis. CB_1_ receptors have been localized in potential generators of migraine pain, like PAG, RVM, and NTC (Moldrich and Wenger, 2000). There are reports that frequency of migraine headache may decrease in persons using medical cannabis (Rhyne et al., 2016). ECs may interact with and modulate several pathways related to migraine, such as opioids, or involved in the mechanism of action of anti-migraine drugs such as triptans (Akerman et al., 2013; Baron, 2015). AEA and other CB agonists have also been demonstrated to inhibit effects on serotonin type 3 receptors, which provide yet another effect when considering that nausea and vomiting are frequent and bothersome accompaniments of migraine (Fan, 1995; Park et al., 2008). CB agonists inhibit the serotonin-induced current in a concentration dependent manner in the rat nodose ganglion neurons by 5-HT3 receptor ion-channel (Fan, 1995). Moreover, they may also act on brain areas involved in emesis, such as the dorsal motor nucleus of the vagus (Van Sickle et al., 2001), where there is a high density of 5-HT3 receptors (Miquel et al., 2002). 5-HT3 inhibition can modulate neurotransmitters, including dopamine, GABA, substance P, and acetylcholine. The anti-migraine effects of the ES are not fully known, although some hypotheses were proposed. Table 1 shows the potential modulatory effects of ECs on migraine pain.

**Table 1:** Potential effects of endocannabinoids on migraine pain.

**Target**	**Effects**	**References**
Trigeminovascular activation	Substance P ↓ CGRP/nitric oxide ↓ Cyclooxygenase ↓ PGE-2 synthesis ↓ glutamate release ↓	Pertwee, 2001; Akerman et al., 2004; Sarchielli et al., 2007; La Rana et al., 2008; Chiou et al., 2013
Serotonergic system	Serotonin release ↓ platelets aggregation ↓ 5-HT2A ↑	Volfe et al., 1985; Ohuoha et al., 1994; Boger et al., 1998; Rossi et al., 2008; Parker et al., 2011; Mendiguren et al., 2018
Brainstem	NF-κB activation ↓ kynurenine pathway modulation	Kelly and Chapman, 2001; Nagy-Grócz et al., 2016
Hypothalamus	Glutamate release ↓	Di et al., 2005
Periaqueductal gray	Proenkephalin expression ↑	Manzanares et al., 1998

Clinical observations show that women migraine without aura or episodic tension-type headache have increased FAAH and endocannabinoid membrane transporter (EMT) activities in platelets, which is consistent with reduced AEA levels (Cupini et al., 2006). In addition, women with episodic migraine have increased CB_1_ receptor binding during the interictal period, as assessed by positron emission tomography; this increase is especially evident in brain regions that exert top-down influences to modulate pain (Van der Schueren et al., 2012). Variants in the CB_1_ receptor gene increase the risk of migraine attack with nausea in life stress exposed subjects (Juhasz et al., 2017). Recently Gouveia-Figueira et al. (2017) failed to detect significant changes in the plasma levels of AEA and other fatty acid ethanolamides between patients with episodic migraine and controls. These contrasting findings may be related to higher inter-subject variability of EC levels in the evaluated cohorts or to a different migraine load on the populations investigated.

More consistent are the findings regarding the involvement of the ES in chronic migraine (CM). Subjects with CM with and without medication overuse headache (MOH) showed reduced activities of FAAH and EMT in platelets when compared to either controls or episodic migraine (Cupini et al., 2008). In another study, 2-AG and AEA platelet levels were significantly lower in MOH and CM patients compared to controls, without significant differences between the two patient groups (Rossi et al., 2008). These findings suggest an adaptive behavior induced by chronic headache *per se*, while medication overuse is apparently not related with EC activity. Interestingly, serotonin levels were reduced in the MOH and CM patients, with lower values detected in females as compared to males (Rossi et al., 2008) and that serotonin levels were also associated with 2-AG tone, with a higher correlation coefficient for MOH patients. This latter finding suggests a possible role for 2-AG, together with serotonin, in the “addiction” aspect of MOH. In this frame, it is worth mentioning that successful detoxification of MOH subjects is accompanied by a reduction in FAAH activity in platelets. This biochemical change is associated with the normalization of neurophysiological parameters that indicated a status of sensitization of the pain pathways (Perrotta et al., 2012). A reduction in AEA and an increase in PEA levels was also found in the cerebrospinal fluid of both CM and MOH patients (Sarchielli et al., 2007), pointing to a central alteration of ES in these subjects.

Inflammation and nerve injury cause changes in local AEA levels (Jhaveri et al., 2007). As mentioned before, AEA is produced on demand during inflammatory conditions and it is rapidly degraded by FAAH activity. Thus, AEA tone can be modulated by FAAH activity in both periphery and CNS. Increased activation of the TS may theoretically lead to reduced levels of AEA, which may, in turn, lead to an increased CGRP and NO release. AEA indeed inhibits the neurogenic dural vasodilatation, as well as CGRP-induced and NO-induced dural vessel dilation (Akerman et al., 2004). The CB_1_ receptor antagonist, AM251, reversed this inhibitory activity, suggesting that CB_1_ receptors may be implicated in the relationship between headache and dural blood vessel dilation and migraine mediators. Cortical spreading depression (CSD) is a self-propagating wave of neuronal hyperexcitability that has a role in migraine (Goadsby, 2007). WIN55,212-2, a CB1 receptor agonist, inhibited the amplitude, duration and velocity of CSD propagation, while JWH 133, a CB2 receptor agonist, was devoid of any effect (Kazemi et al., 2012).

The trigeminal firing in the trigeminocervical complex induced by AEA inhibition is reversed after CB_1_ receptor antagonism, thus suggesting that the central effects of AEA are principally CB_1_-mediated (Akerman et al., 2007). CB_1_ receptor activation in the ventrolateral PAG, obtained with the administration of WIN55,212-2, attenuates the activity evoked by dural stimulation in A-fiber neurons and the basal spontaneous activity in the trigeminocervical complex of rodent. These findings suggest that, in the brainstem, ECs may provide to descending modulation upon basal trigeminovascular neuronal tone and A-fiber dural-nociceptive responses, (Akerman et al., 2013). Changes in FAAH and MGL activities were found in the brainstem and hypothalamus of rats treated with nitroglycerin (NTG) (Greco et al., 2010b), a recognized animal model of migraine (Buzzi and Tassorelli, 2010). NTG in rat causes an increased sensitivity to nociceptive tests and c-fos protein expression in brain areas nuclei involved in migraine pain transmission, such as NTC (Greco et al., 2011a). The use of this model by us and other groups has allowed the in-depth exploration of the mechanisms underlying the modulation of the ECs and the nociceptive activation of the TS during a migraine attack. In particular, we reported an increased FAAH activity in the hypothalamus and in the medulla area, where NTC neurons are located, and an up-regulation of CB_1_ receptor binding sites in the same areas (Greco et al., 2010b), suggesting a key role of AEA in the cephalic pain.

Our findings also show that AEA pretreatment significantly reduces NTG-induced behavioral nocifensive and NTG-induced neuronal activation in the NTC (Greco et al., 2011a); moreover, AEA may modulate central sensitization through TRPV1, COX2 expression and NF-κB inhibition in NTC (Nagy-Grócz et al., 2016). The CB2 receptors activation in pain modulation has been considered in the past, showing analgesic activity in several models of pain (Nackley et al., 2003, 2004; Quartilho et al., 2003). In our migraine model, we have also shown that CB_2_ receptor activation significantly decreases nocifensive behavior of rats made hyperalgesic by NTG (Greco et al., 2014). Likewise, MGL inhibition, and the subsequent increase in central and/or peripheral levels of 2-AG, reduces NTG-induced hyperalgesia at the nociceptive tests, and attenuates c-Fos protein expression in brain areas implicated in the transmission or integration of cephalic pain (Greco et al., 2017).

## Recent advances on FAAH inhibition in migraine pain

Though the analgesic effects of cannabinoids are fairly well established, their use in therapy remains limited by their psychoactive properties (Borgelt et al., 2013). Recent safety concerns about FAAH inhibitors turned out to be ungrounded, and due to off-target effects. Clearly, the successful development of compounds that modulate ECs tone for the pain relief in humans will hinge on the ability to separate psychotropic effects from therapeutic ones, and to control for potential off-target interactions. Positive allosteric modulation of CB_1_ receptor signaling may represent a safe analgesic alternative strategy that lacks tolerance, dependence and abuse liability (Khurana et al., 2017; Slivicki et al., 2017). Several studies show that also increasing ECs levels through the inhibition of catabolic enzymes, FAAH in particular, would decrease cannabimimetic side effects (Piomelli et al., 2006; Booker et al., 2012).

Besides AEA, FAAH degrades other fatty acid amides, which have several biological functions and mechanisms of action (Ahn et al., 2008). FAAH is contained in intracellular membranes of postsynaptic somata and dendrites of the mammalian brain (Gulyas et al., 2004). In many cerebral structures FAAH and CB_1_ receptors cellular co-localization in cell bodies or dendrites in proximity of CB_1_-expressing fibers (Egertová et al., 1998). Manipulations of full-length and transmembrane-truncated FAAH variants have offered a characterization of mechanisms of action (McKinney and Cravatt, 2005). In particular, these studies showed that, unlike most serine hydrolases, which use a histidine residue as a catalytic base, FAAH recruits a lysine to hydrolyze both amides and esters at equivalent rates (Patricelli and Cravatt, 1999). Numerous FAAH inhibitors have been developed and tested in animal models of disease (Jayamanne et al., 2006; Kinsey et al., 2009). In particular, the FAAH inhibition induces anti-inflammatory effects *in vivo* (Jayamanne et al., 2006; Booker et al., 2012; Wilkerson et al., 2017). In addition, mutant mice for FAAH enzyme in non-neuronal cells, but with FAAH activity conserved in peripheral and central neurons, have a phenotype in which basal nociceptive transmission is connected to the reduced responsiveness to pro-inflammatory mediators (Cravatt et al., 2004). Researchers suggest that AEA regulates nociceptive transmission primarily at the peripheral level (Calignano et al., 1998; Clapper et al., 2010; Piomelli and Sasso, 2014).

Numerous studies have shown that FAAH inhibition causes analgesia and reduces inflammation in animal models of acute inflammatory pain (Kinsey et al., 2010; Lodola et al., 2015; Nasirinezhad et al., 2015), but there is little information on their effects in migraine. Recently, it was reported that AEA modulates the analgesic activity in the orofacial area and that endomorphin-2-induced antinociception is mediated by μ and CB_1_ receptors (Zubrzycki et al., 2017). Nozaki et al. (2015) demonstrated that NTG-induced mechanical allodynia and c-Fos protein in the NTC is abolished in FAAH-deficient mice or after URB597 treatment, a global FAAH inhibitor, via maintenance of central and peripheral AEA levels. When considering that NTG is thought to activate meningeal trigeminovascular terminals via the local NO formation (Reuter et al., 2001; Greco et al., 2011b), it is probable that URB597 interferes with this mechanism of peripheral sensitization. Accordingly, we have shown that a peripherally restricted FAAH inhibitor, the compound URB937, inhibits NTG-induced nocifensive behaviors (plantar and orofacial formalin test, tail flick test), neuronal activation in the NTC and locus coeruleus (Greco et al., 2015). In agreement with these data, URB937 decreases the c-Fos expression induced by plantar formalin injection in spinal cord regions involved in nociceptive processing by the CB_1_ receptors (Clapper et al., 2010).

Thus, since URB937 acts only peripherally, it seems reasonable to hypothesize that its mechanism of action relies on the maintenance of higher levels of AEA released by nervous terminal located in the injured peripheral tissues (hindpaw, upper lip, tail) (Agarwal et al., 2007) or in the dura, with consequent CB_1_ receptor activation in trigeminovascular endings. An additional mechanism, is probably represented by the blockade of NTG-induced inflammatory pathway mediated by NO in dura mater and/or trigeminal ganglia. In agreement with this hypothesis, *in vitro* studies have shown that increased AEA tone, through the inhibition of its degradation or uptake, decreases the cytokines and NO levels (Correa et al., 2009, 2010).

## Outlook

Pain is a heterogeneous condition and it should be treated as such. With its lack of sensitivity to standard analgesic medications (Ong and De Felice, 2017), migraine pain is a case in point and—perhaps better than most other forms of pain—underscores the need for tailored therapies. The human data and preclinical studies reviewed here confirm the importance of FAAH-regulated AEA signaling in the processing of nociceptive signals outside the CNS (Greco et al., 2010a; Piomelli and Sasso, 2014) and specifically point to peripheral FAAH inhibition as a possible therapeutic opportunity for migraine pain. Future experiments should be aimed at unlocking the precise cellular mechanisms and neural circuits through which peripheral FAAH blockade exerts its analgesic effects in migraine pain, further exploring the ground for potential clinical trials.

## Author contributions

RG: designed this review; CD and AZ: contributed to cited data from our group; DP and CT: revised the final version of the manuscript.

### Conflict of interest statement

The authors declare that the research was conducted in the absence of any commercial or financial relationships that could be construed as a potential conflict of interest.
